# German Translation and Cross-Cultural Adaptation of the STarT Back Screening Tool

**DOI:** 10.1371/journal.pone.0132068

**Published:** 2015-07-10

**Authors:** Bernhard Aebischer, Jonathan C. Hill, Roger Hilfiker, Sven Karstens

**Affiliations:** 1 Health Division, University of Applied Sciences, Bern, Switzerland; 2 Institute for Primary Care and Health Sciences, Keele University, Staffordshire, Great Britain; 3 School of Health Sciences, University of Applied Sciences and Arts Western Switzerland Valais, Wallis, Switzerland; 4 Department of General Practice and Health Services Research, University Hospital, Heidelberg, Germany; Iranian Institute for Health Sciences Research, ACECR, IRAN, ISLAMIC REPUBLIC OF

## Abstract

**Background:**

Although evidence based treatment approaches for acute low back pain are available, the prevention of persistent disabling symptoms remains a challenge. Subgroup targeted treatment using adequate screening tools may be a key component for the development of new treatment concepts and is demonstrating promising early evidence. The Keele STarT Back Screening Tool is a practical instrument, developed to stratify patients with back pain according to their risk of persistent disabling symptoms. The aim of this study was to translate and cross-culturally adapt the STarT tool into German (STarT-G) and to investigate its psychometric properties.

**Methods:**

The translation was performed according to internationally accepted guidelines and pretested to assess face validity among patients. Psychometric testing was then performed within a cross-sectional cohort of adult patients attending physiotherapy practices for back pain. Patients completed a booklet containing STarT-G and 5 reference standard questionnaires. Measurement properties of the STarT-G were explored including construct validity, floor and ceiling effects, and discriminative abilities.

**Results:**

The pretests (n=25) showed good face validity including strong comprehension and acceptability of the STarT-G with only item 5 (fear avoidance) manifesting some ambiguities. The questionnaires were sent to 74 and completed by 50 patients (68%) of whom mean age was 46 (SD 14.5) years and 52% were male. Spearman’s rank correlations for construct validity ranged from 0.35 to 0.56. AUCs for discriminative ability ranged from 0.79 to 0.91. Neither floor nor ceiling effects were observed. There were 28 (57%) participants defined as low risk, 17 (35%) as medium risk, and 4 (8%) as high risk.

**Conclusion:**

STarT-G is linguistically valid for German speaking countries. For the selected population, the correlations indicate acceptable validity and AUC showed satisfying discrimination. Data for psychometric properties have to be confirmed in a large scale study with a representative sample.

## Introduction

Low back pain (LBP) has a high prevalence and incidence in Switzerland [1] and Germany [2] with estimates suggesting a 4 week prevalence of 43% among the Swiss population in 2007 [3]. Of all back pain patients with acute problems, 6 to 17% are sliding into chronicity [4] [5]. These problems frequently lead to a reduction in patients’ quality of life and working ability. For society the economic burden due to back pain is large with costs for treatment and work absence estimated as € 2.4 to 2.9 billion in Switzerland [6].

In clinical practice, the term ‘low back pain’ covers a broad set of symptoms and behaviors rather than a single clinical condition. This inherently means that patients with LBP are a heterogeneous group requiring different management approaches despite a range of effective treatments being available [7] [8]. As a consequence, even though patients present with essentially the same site of pain, each have individual biomedical features, psychosocial and prognostic profiles that influence decisions regarding their management plan [9]. For example, for some low risk patients providing advice and easily applied self-care exercises will be sufficient to resolve the problem, while for others who are at high risk of persistent disability, psychological distress may be associated with depressed mood and limitation of activities, influencing recovery. A challenging task for clinicians is therefore to distil from individual patients the right treatment approach for that individual the first time they consult. As a result many clinicians use a ‘wait and see’ approach, beginning with, for example, low level analgesia before progressing patients onto more costly treatment services such as referral to physiotherapy for additional support.

The Keele STarT (Subgroup Targeted Treatment) Back Screening Tool was designed as a subgrouping tool to help guide first contact clinicians in their initial back pain assessment process by identifying an individual’s overall risk status for persistent disabling pain, based on specific prognostic factors that might be appropriate treatment targets in their management [10] [11]. A number of different approaches and methods to subgroup patients with low back pain have been established [12] [13]. The STarT Back Screening Tool is a part of a stratified care approach that is supported by evidence from a high quality randomized controlled trial [14]. The tool was developed and validated by a research team in England [15] for use in general practice and allows a fast, easy-to-use and reliable assessment of low back pain prognosis. It consists of 9 items, of which 8 have dichotomous “yes” or “no” response options with a ninth item using a 5-point Likert response scale. Items 1 to 4 relate to physical aspects of low back pain, whilst items 5 to 9 explore psychosocial risk factors and therefore form a psychosocial subscale. Using established scoring thresholds the instrument is used to discriminate patients who are at low, medium or high risk for chronicity (http://www.keele.ac.uk/sbst/). In addition to the risk assessment, the STarT Back stratified care approach suggests matched treatment pathways for each of the risk groups. Thus the practitioner gains not only a valid prognostic tool to support decision making, but is provided with practical evidence-based treatment options that are matched to the prognostic profile of the individual.

The STarT tool has been translated into over 20 languages. Despite steadily increasing interest in clinical practice and in the scientific community among German speaking countries, to date only a simple translation into German exists.

The aim of this study was therefore, to formally translate and cross-culturally adapt the STarT Back Screening Tool into German and to give initial information on construct validity, discriminative ability, and floor or ceiling effects.

## Methods

### Translation and cross-cultural adaptation

The translation and cross-cultural adaptation was done according to internationally accepted guidelines [16] with permission for translation from the original STarT developers. The translation committee consisted of five people. The three forward translators with German mother tongue included a German physiotherapist (informed about the project), a non-medical Swiss individual and an informed German Master of Science in Public Health. They were asked to note any remarks and questions while translating. The three forward translations were synthesized into a final forward version by the German speaking members of the steering committee, discussions were held via email and telephone. Three rounds were needed to reach overall agreement. The backward translations were done by two non-medical professional translators who were native speakers of English, recruited through a professional translation office. The two backward translations were synthesised by the first author and sent for discussion to the developer of the original English version. All translators worked independently from each other.

To check for acceptability and comprehension a pre-test was carried out with 25 patients from two private physiotherapy practices (15 in Switzerland, 10 in Germany). Eligibility criteria were age >18, low back pain, able to fill out the questionnaire on their own, and a native German speaker. While the tool was completed, observations were made of any hesitations or comprehension difficulties. Patients were also asked open questions to determine if they experienced any problems with the tool and their answers noted. Findings were discussed with the developer of the English version.

### Study design for psychometric properties

Patients with back pain were recruited through private physiotherapy practices and hospital physiotherapy departments in German speaking Cantons in Switzerland. All volunteering physiotherapists in these practices and departments were members of the Swiss physiotherapy association and contacted as such via the association’s email list. Inclusion criteria were low back pain no longer than 6 months, normal cognitive functions, age older than 18 years and German speaking. Exclusion criteria were: previous surgical interventions, previous acute trauma, tumors or cauda equina syndrome (“red flags”). Patients’ addresses were sent to the study coordinator (BA) who in turn mailed the study material as described below within 48 hours.

Reference standard questionnaires (RSQ) were the same as in the original STarT development study to allow comparison [15]. Disability was captured using the Roland and Morris Disability Questionnaire (RMDQ) [17], fear avoidance beliefs were measured using the Tampa Scale of Kinesiophobia (TSK) [18], catastrophizing with the Pain Catastrophizing Scale (PCS) [19], depression and anxiety with the Hospital Anxiety and Depression Scale (HADS) [20] and the 2 Item—Patient Health Questionnaire (PHQ-2) [21]. A composite reference standard was determined which defined patients as being ‘distressed’ if they were simultaneously above cut-off thresholds in the following three psychosocial measures, TSK, PCS and PHQ-2 (for cut-off values see Table 1). With exception of the TSK (officially translated but not yet validated), validated German versions of all questionnaires were used [17] [19–21]. Patients were sent a participant information sheet, informed consent form, a booklet containing the STarT-G, five reference standard questionnaires (RSQ), single item questions for episode duration, pain intensity, referred leg pain and comorbid musculoskeletal pain, and a prepaid reply envelope. Patients were asked to fill in and return the questionnaires as soon as possible. Follow up reminders were made by letter and/or by phone after 3 to 4 weeks.

**Table 1:** Cut-off values for reference standard questionnaires.

RSQ	Set cut-off value	Article
**TSK**	≥ 41^&^	Nigbur et al. [18]
**PCS**	≥ 20^&^	Meyer et al. [30]
**PHQ-2**	≥ 3	Löwe et al. [21]
**HADS**	Anxiety ≥ 11	Herrmann-Lingen et al. [31]
	Depression ≥ 9	
**RMDQ**	≥ 6^£^	Exner and Keel [32]
**VAS**	≥ 50^£^	Jensen et.al. [33]

RSQ: reference standard questionnaire, TSK: Tampa Scale of Kinesiophobia, PCS: Pain Catastrophizing Scale, PHQ-2: Patient Health Questionnaire– 2 items version, HADS: Hospital Anxiety and Depression Scale, RMDQ: Roland Morris Disability Questionnaire, VAS: Visual Analogue Scale.

^&^ No cut-off value for German population available,

^£^ median utilized as cut-off.

### Ethics Statement

The responsible Cantonal Ethical committees were contacted. According to this body formal approval was not necessary as the study did not involve a change to usual treatment. All patients received information about the study by their physiotherapists and gave written informed consent to participate.

### Statistics

Descriptive statistics were produced for participant’s baseline characteristics, together with data about drop-outs and missing data. The choice of statistical tests for hypothesis testing was made according to instrument psychometric testing guidelines by Terwee et al [22]. Statistical significance was calculated with one-tailed probability with a significance level of p < 0.05.

In order to examine construct validity, Spearmans coefficients were calculated and the descriptions used to explain the magnitude of these correlations followed those used by the original tool developers [15]. In addition to correlations between the RSQ and the STarT-G total and subscore respectively, correlations were also examined between the risk subgroup level and each RSQ. Corresponding to the approach of the original authors, boxplots were constructed to visualize the correlations between total score and RMDQ and psychosocial subscore against PCS.

Discriminative ability was assessed by calculating area under the curves (AUCs). Adjectives that can be used to describe increasing AUCs have been proposed by Hosmer and Lemeshow [15] with an AUC = 0.5 suggesting ‘no discrimination’, 0.7 to < 0.8 considered ‘acceptable discrimination’, 0.8 to 0.9 considered ‘excellent discrimination’ and >0.9 considered ‘outstanding discrimination’. Discriminative ability of the total tool score for disability was chosen to be consistent to the original study to assist interpretation of findings.

Floor and ceiling effects were defined as present if more than 15% of the responders achieved the lowest or highest possible STarT-G total score [22]. Analysis was performed using SPSS version 20.0.

Terwee et al. [22] suggest that an appropriate sample size of at least n = 50 patients is required for assessment of construct validity, reliability, floor and ceiling effects and interpretability. This number was therefore chosen as the minimal sample size.

## Results

### Translation and pretesting

The translation process was conducted as planned. The synthesized forward and backward translated versions were agreed by members of the German and English speaking expert panel, respectively. The backwards translation showed good accordance with the original English version. The linguistic analysis showed that there are no Helvetisms [23]. The pretest showed good acceptability and comprehension except for item 5 (fear of movement) which did have some comprehension problems. The questionnaire can be obtained from the authors via email.

### Psychometrics

Between September 2012 and February 2013, 35 physiotherapists from 24 private practices and physiotherapy out-patient departments in hospitals and clinics in German speaking Switzerland recruited 74 patients for the study. The study material was send to the patients per post. Fifty patients returned a completed questionnaire booklet (40 from private practices, 10 from out-patient departments). Of the responders 52% were men and the mean age was 46 (SD 14.5). Further baseline characteristics are shown in Table 2. Non-responders were 58% male. Missing data was found in only 3 questionnaires (6%): 1 missing the VAS pain intensity item, 1 missed RMDQ and TSK, and 1 filled out only the single questions and the HADS.

**Table 2:** Baseline characteristics.

	STarT-G	STarT
**n**	50	131
**Gender female (%)**	48	60
**Mean age (SD)**	46 (14.5)	44 (10)
**STarT-Score**		
**Mean total Score (SD)**	3.5 (1.9)	4.4 (2.6)
**Mean subscore (SD)**	1.5 (1.3)	2.2 (1.6)
**STarT risk group**		
**Low risk (%)**	57	41
**Medium risk (%)**	35	34
**High risk (%)**	8	25
**Comorbid pain (%)**	42	48
**Referred pain (%)**	58	57
**Mean TSK (SD)**	34.5 (6.8)	40.8 (7.6)
**Mean PCS (SD)**	18.6 (10.2)	18.9 (12.5)
**Mean RMDQ (SD)**	6.4 (4.4)	8.6 (6.6)
**Mean PHQ-2 (SD)**	1.9 (1.5)	NA
**Pain duration**		
**< 1 month (%)**	18	25
**1–3 month (%)**	34	15
**4–6 month (%)**	8	12
**>6 month (%)**	40	48
**HADS**		
**Mean anxiety (SD)**	6.1 (3.8)	NA
**Mean depression (SD)**	4.0 (2.6)	NA
**Pain**		
**Mild (0–5; %)**	55	65
**Moderate (6–7; %)**	35	22
**Severe (8–10; %)**	10	13

STarT-values see Hill [15]; RSQ: reference standard questionnaire, TSK: Tampa Scale of Kinesiophobia, PCS: Pain Catastrophizing Scale, PHQ-2: Patient Health Questionnaire– 2 items version, HADS: Hospital Anxiety and Depression Scale, RMDQ: Roland Morris Disability Questionnaire, VAS: Visual Analogue Scale.

**Fig 1:**
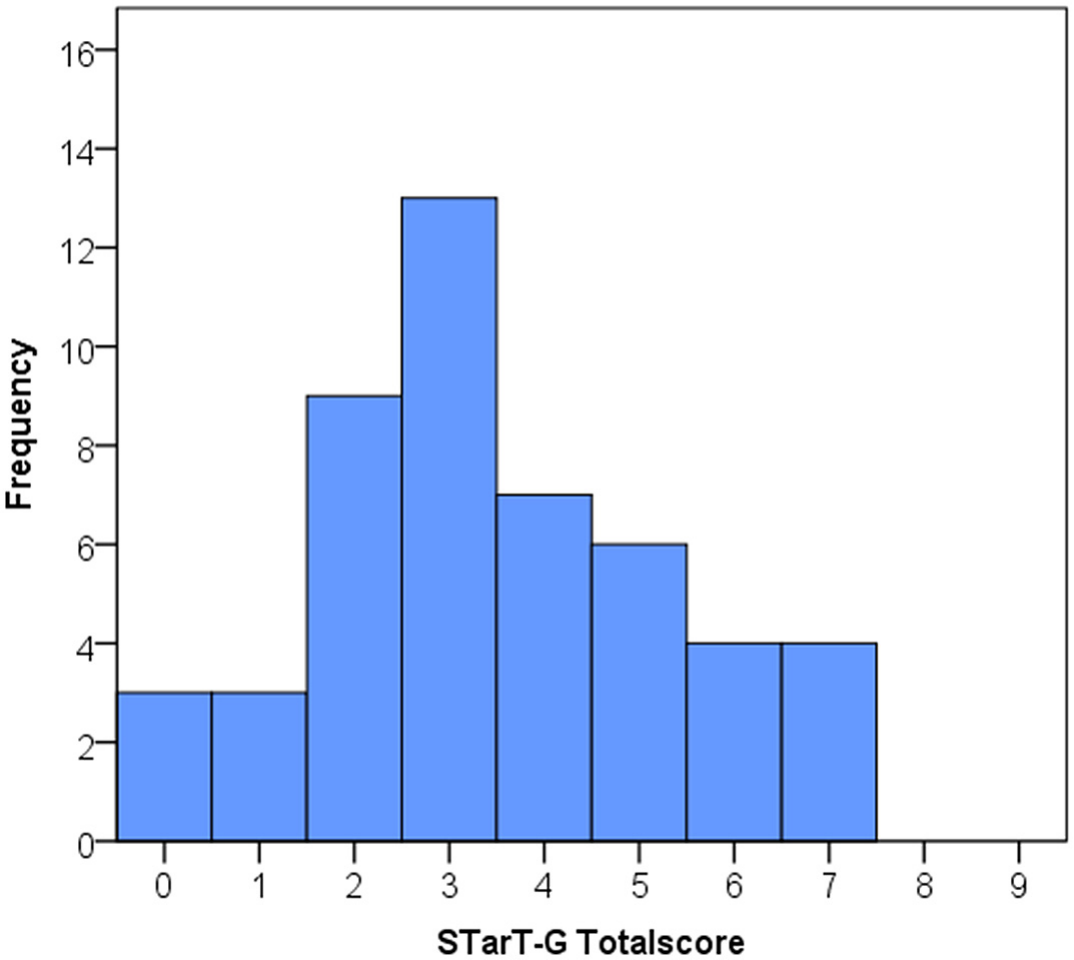
Distribution STarT-G totalscore.

**Fig 2:**
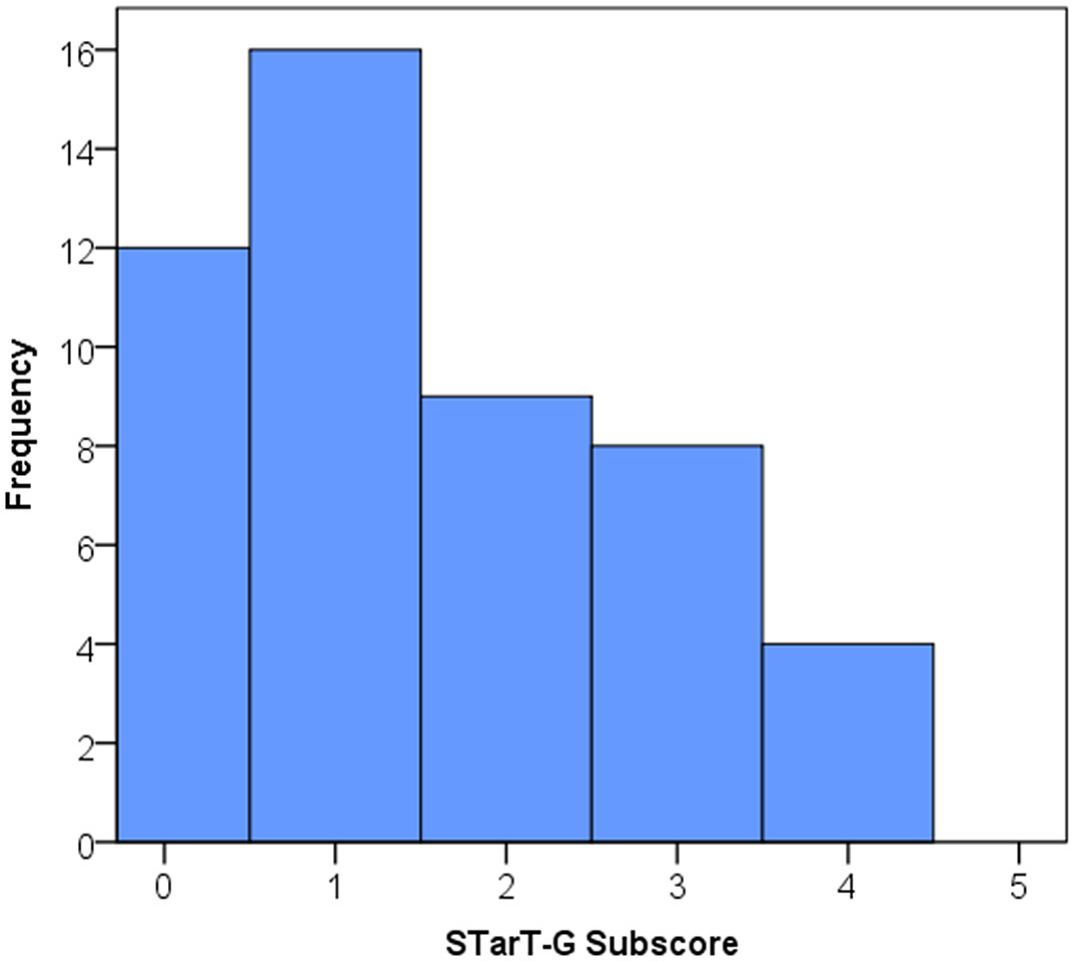
Distribution STarT-G psychosocial subscore.

The distribution across the prognostic subgroups was: 28 patients low risk, 17 medium risk, and 4 high risk. STarT-G total score and subscore means (SD) were 3.5 (SD 1.9) and 1.5 (SD 1.3) respectively. Figs 1 and 2 show a histogram displaying the STarT-G total and subscore distributions. Scores of the reference standard questionnaires are shown in Table 2. The distribution of positive responses to each STarT item is shown in Table 3.

**Table 3:** Comparison of positive answers to StarT items (“Agree” / “Trifft zu”).

Item	STarT-G (%)	STarT (%)
**1 Referred pain**	51	58
**2 Comorbid pain**	39	48
**3 Walk short distances**	47	48
**4 Dress slowly**	59	64
**5 Fear**	20	51
**6 Anxiety**	49	52
**7 Catastrophizing**	8	45
**8 Depression**	31	59
**9 Very / extremely bothered**	43	43

STarT-values see Hill [15].

Statistically significant Spearmans rank correlation coefficients were observed, ranging from 0.35 to 0.56 (Table 4), demonstrating moderate to large convergent construct validity. The resulting coefficients were 0.55 (95% CI; 0.30–0.74) for the correlation of the STarT-G total score with disability (RMDQ), 0.46 (0.17–0.68) for the psychosocial subscore with fear (TSK), 0.37 (0.10–0.62) with catastrophizing and 0.54 (0.29–0.72) with depression.

**Table 4:** Convergent construct validity: Spearmans rank correlation coefficients.

RSQ	Total score		Psychosocial Subscore		Risk group
	STarT-G (95%CI)	STarT	STarT-G (95%CI)	STarT	(95%CI)
**TSK**	0.40** (0.13,0.63)	0.60	0.46** (0.17,0.68)	0.66	0.48** (0.22,0.70)
**PCS**	0.27* (-0.06,0.54)	0.57	0.37** (0.10,0.62)	0.68	0.15 (-0.17,0.47)
**HADS anx**	0.41** (0.15,0.62)	—	0.54** (0.32,0.70)	—	0.20 (-o.12,0.49)
**HADS depr**	0.26* (-0.06,0.52)	—	0.35** (0.06,0.61)	—	0.18 (-0.11,0.45)
**PHQ-2**	0.55** (0.30,0.74)	0.42	0.54** (0.29,0.72)	0.51	0.56** (0.31,0.74)
**RMDQ**	0.55** (0.30,0.73)	0.81	0.46** (0.21,0.64)	0.78	0.45** (0.17,0.68)
**VAS**	0.47** (0.18,0.70)	—	0.46** (0.17,0.68)	—	0.39** (0.10,0.62)

STarT-values see Hill [15].

* Statistically significant on level 0.05 (t-test),

** significant on level 0.01.

RSQ: reference standard questionnaire, TSK: Tampa Scale of Kinesiophobia, PCS: Pain Catastrophizing Scale, PHQ-2: Patient Health Questionnaire– 2 items version, HADS anx/depr: Hospital Anxiety and Depression Scale (anxiety/depression), RMDQ: Roland Morris Disability Questionnaire, VAS: Visual Analogue Scale.

**Fig 3:**
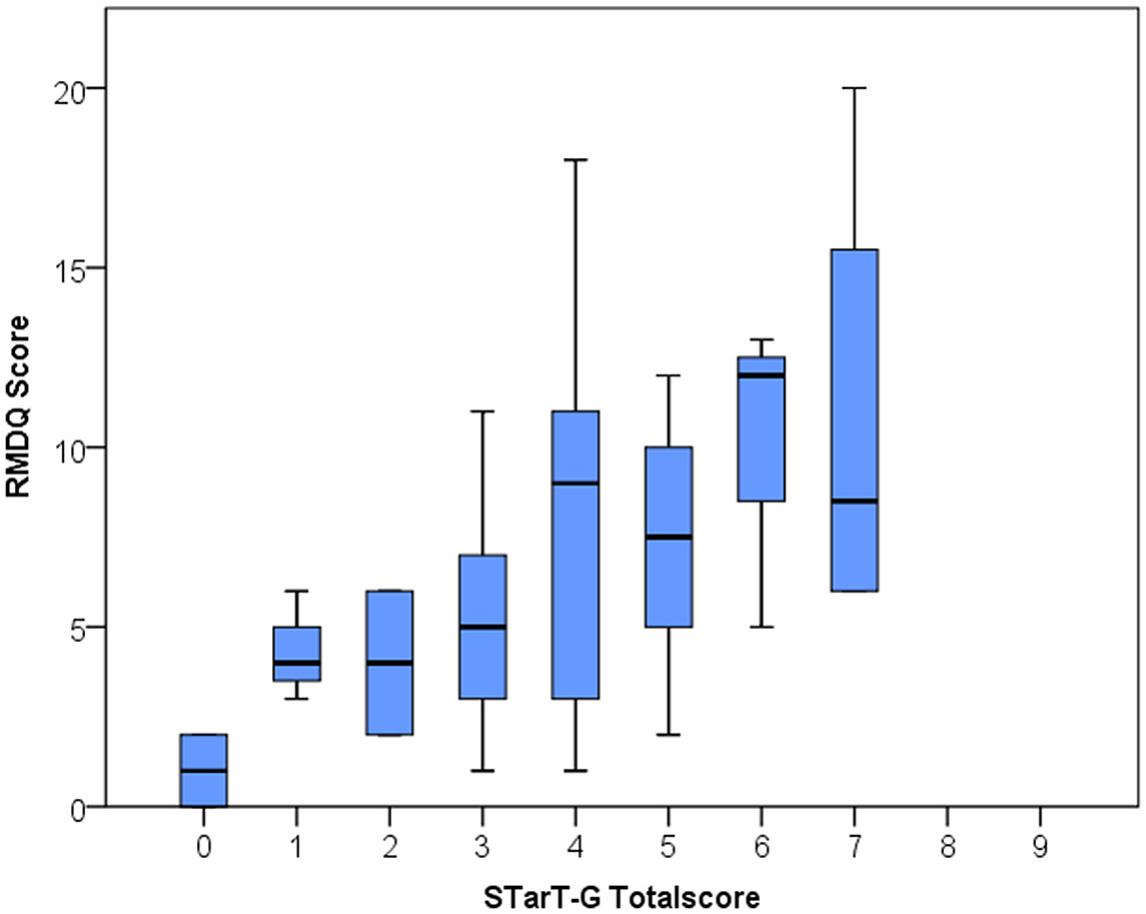
STarT-G totalscore against RMDQ Score. RMDQ: Roland Morris Disability Questionnaire.

**Fig 4:**
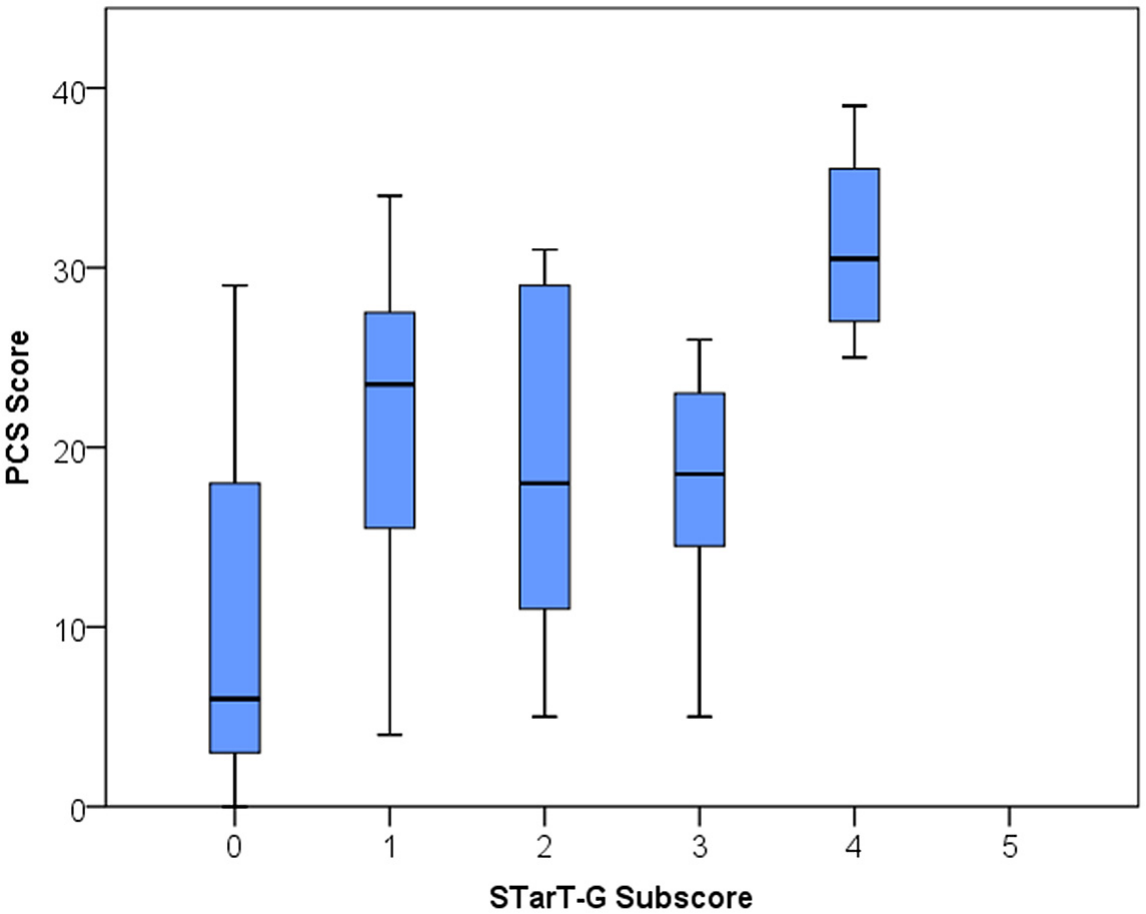
STarT-G psychosocial subscore against PCS Score. PCS: Pain Catastrophizing Scale.

Consistent with the original STarT developers [15], box plots for STarT-G total scores against RMDQ scores and psychosocial subscale scores against the PCS are shown in Fig 3 and Fig 4.

**Fig 5:**
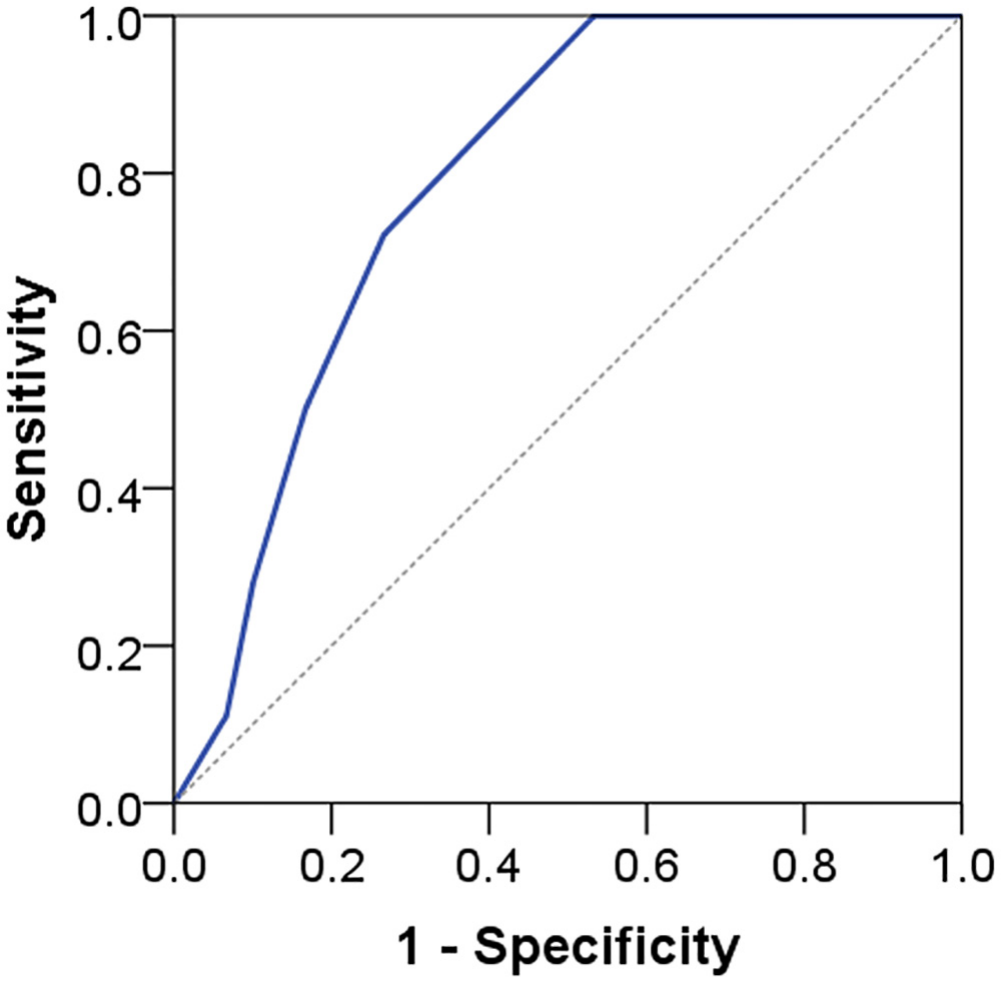
Receiver operating characteristic curve for STarT-G totalscore against RMDQ cases. AUC 0.79 (95% CI 0.67–0.92); RMDQ: Roland Morris Disability Questionnaire.

**Fig 6:**
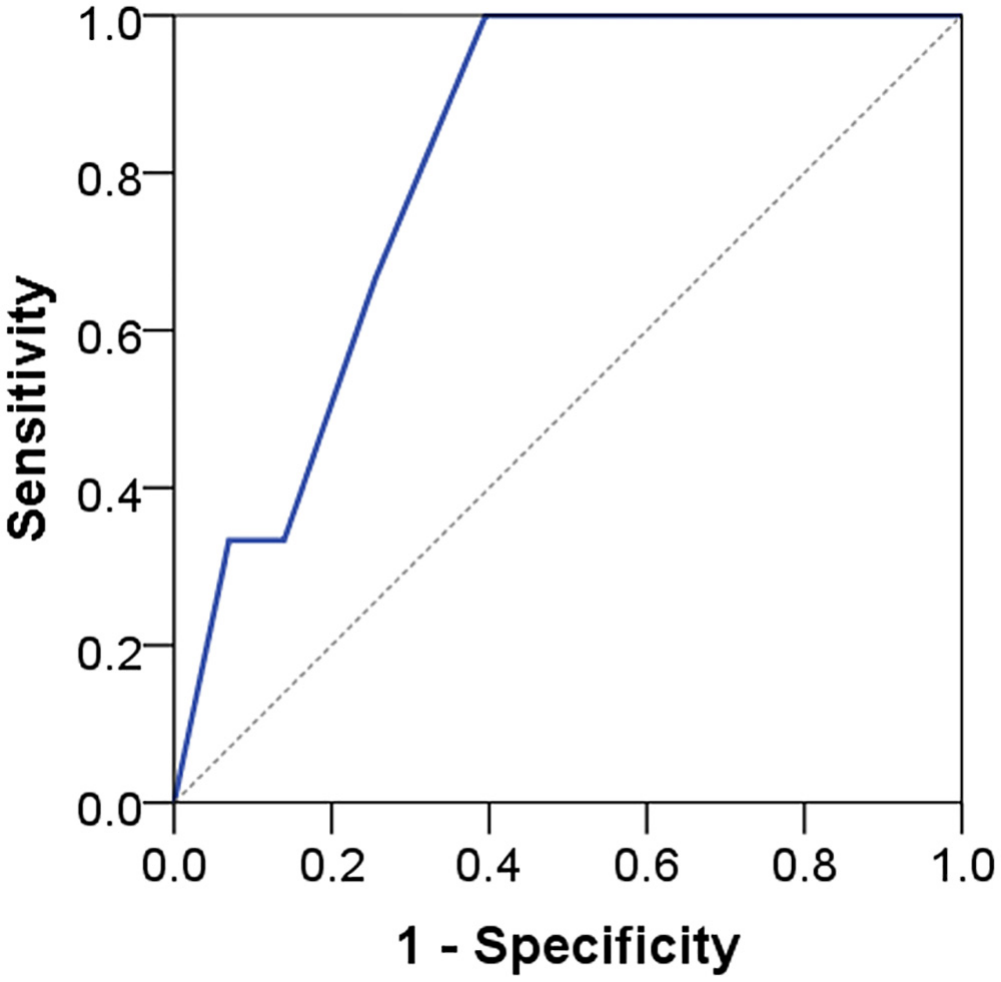
Receiver operating characteristic curve for STarT-G totalscore against composite reference standard cases. AUC 0.81 (95% CI 0.65–0.98).

**Fig 7:**
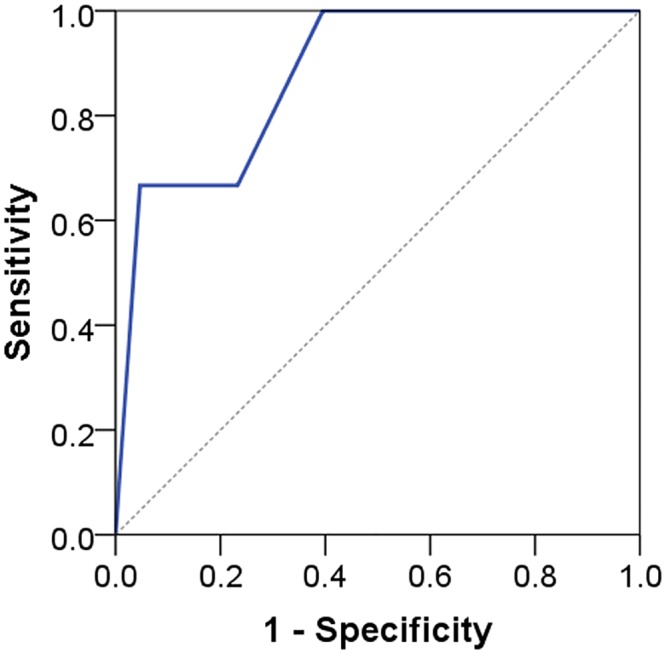
Receiver operating characteristic curve for STarT-G psychosocial subscore against composite reference standard cases. AUC 0.88 (95% CI 0.71–1.00).

**Fig 8:**
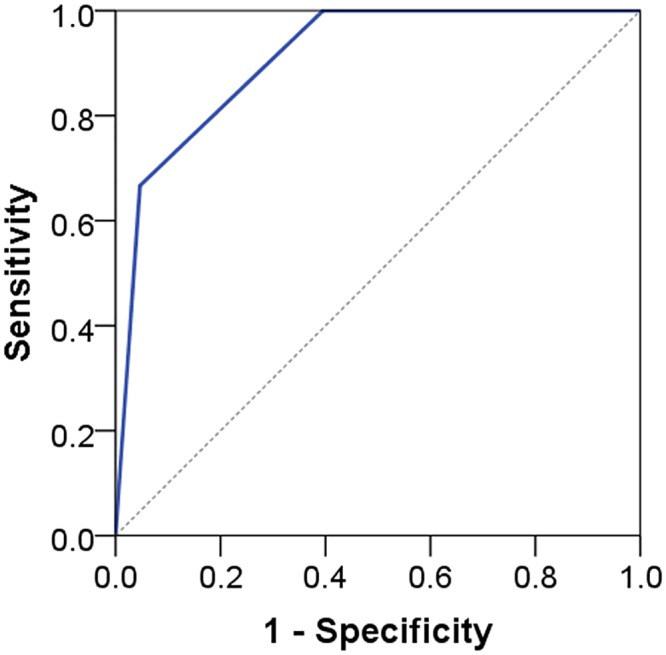
Receiver operating characteristic curve for risk group against composite reference standard cases. AUC 0.91 (95% CI 0.77–1.00).

AUC ranged from 0.79 to 0.91 indicating acceptable to outstanding discrimination (see Figs 5 to 8). No floor or ceiling effects were observed as 6.1% of the patients had a total score of 0, and none had scores of 8 or 9 points.

## Discussion

We translated and cross culturally adapted the STarT-tool into German and gathered first information on its psychometric properties. Overall, the pretest results indicate translated items were well understood and acceptable. The STarT-G also demonstrated acceptable to outstanding discriminative ability and moderate to large convergent construct validity.

### Cross cultural adaptation

A strength of the cross cultural adaptation process was the strict adherence to established guidelines [16]. Following the defined process, we developed a comprehensible German version of the STarT–the STarT-G. During the pretests only for item 5 some patients hesitated while answering. A reoccurring theme from patients was an uncertainty about the meaning of “körperlich aktiv” (“physically active”) and its relation to any kind of physical activities or only to occupational activities. The fact that in both the Danish translation [24] and in the English original study [15] comparable problems occurred, supports the assumption that the translation was sufficient and the reason for the uncertainty of the patients may be an issue inherent with the original item used. Since the pretests were conducted in both Germany and Switzerland with positive results and the linguistic analysis for Swiss idiosyncrasies resulted in no ‘Helvetisms’, the questionnaire can be assumed to be linguistically valid for German speaking countries.

### Psychometric testing

The mailing to private practices and physiotherapy outpatients departments in hospitals and clinics enabled the inclusion of the minimal sample size suggested by Terwee et al. [22]. Having only the mail addresses of the non-responders it was not possible to analyze differences in comparison to responders. While the demographics differed in gender but not in age from the original STarT-study [15], there were distinct differences in risk group distribution between the studies with the English sample having more high risk and less low risk patients. Furthermore, scores for reference standard questionnaires and the distribution of positive responses to the single questions differed. An explanation for these findings might be the fact that in contrast to the original study the patients possibly received physiotherapeutic treatment before filling out the questionnaires. Cultural differences between the English and the German Swiss population could be another reason for the lack of high risk patients, in a similar way as Morsø et al. suspected in their study [24].

One positive finding for the STarT-G is that is has strong discriminative ability as the AUCs were high and comparable to the original study. In addition, the very high AUC of risk group against distressed cases (“composite reference standard cases”) demonstrates the justified claim of STarT-G to discriminate between risk groups.

Especially for convergent construct validity our study showed psychometric differences between STarT and STarT-G. There could be a number of reasons for this. First, with the patients receiving physiotherapeutic treatment STarT-G was tested with a specific population different from the one in the original study. Second, within the cohort for this study there was a considerably smaller proportion of high risk patients than in the validation study for the original version [15]. This situation may have lowered the variation in the sample, therefore lowering the covariance and consequently the correlation coefficient. Neither floor nor ceiling effects were present. While the result for the floor effect seems valid, that for the ceiling effect has to be taken with caution because of the few high risk patients.

### Strength and weaknesses

Psychometric testing was orientated on the guideline given by Terwee et al. [22]. Although our sample size fits the suggested of n = 50 our confidence intervals were wide. Due to the pragmatic recruitment it was not possible to control if the participating physiotherapists invited patients at random to participate and at which point of time they filled out the questionnaires. For all 50 responders there was a time span of at least 10 days between collection of the patients address through the recruiting physiotherapists and questionnaire completion. The physiotherapeutic treatment within this period might have influenced not only biomedical but also psychosocial factors [7] and therefore may have affected STarT-G values and the RSQ and thus, led to a reduction of high risk patients. Nevertheless, since it was not the aim of our study the treatment content was not documented. Additionally, the recruitment method didn’t allow controlling neither for the physiotherapist’s selection of patients except for the inclusion and exclusion criteria nor for the frequency or content of the applied physiotherapy. To address the described points a large scale study will be undertaken which will additionally determine reliability coefficients.

### Clinical implications

The STarT-tool was translated to support the management of low back patients in primary care in German speaking areas. Hill et al. demonstrated the usefulness of the instrument in a physiotherapeutic setting and its potential for cost savings and better cost-effectiveness [14]. Based on a population-based cohort study Foster et al. confirmed significant improvements of disability without an increase of health care costs [25]. Despite the need for further evidence for the usefulness of stratified care [26] [27], patient-centered and subgroup-oriented care is generally achieving consensus within the research community as a beneficial direction for clinical practice [28]. As part of the implementation of stratified care into clinical practice in the UK, Hill et al. [14] are training physiotherapists to use the tool appropriately and manage patients according to their matched treatment pathways. For effective implementation of the STarT-G in German speaking areas a comparable training programme may also be required. Moreover education among general physicians to inform them about the helpfulness and relevance of STarT-G is needed [29] in order to ensure appropriate early decision making and to improve suitability of referrals to ongoing treatment such as physiotherapy. A study to explore barriers and enablers for implementation among a German primary care population is currently under way.

## Conclusion

STarT-G is linguistically valid for German speaking areas. Our preliminary results show appropriate convergent and discriminative validity for the tested population. A subsequent study will be undertaken to supplement psychometric properties with a large and representative sample.
